# Study on the synergies and trade-offs of sectoral water use and their relationship with economic development

**DOI:** 10.1371/journal.pone.0327626

**Published:** 2025-07-03

**Authors:** Jianqin Ma, Shuoguo Yang, Qing Wu, Xiuping Hao, Bifeng Cui, Zhihong Su

**Affiliations:** 1 School of Water Conservancy, North China University of Water Resources and Electric Power, Zhengzhou, China; 2 College of Surveying and Geo-informatics, North China University of Water Resources and Electric Power, Zhengzhou, China; 3 Henan Key Laboratory of Water Pollution Control and Rehabilitation Technology, Henan University of Urban Construction, Pingdingshan, China; Shandong University, CHINA

## Abstract

Water scarcity is dynamic and complex, beyond geophysical stressors and responses, it is critical to also consider how multi-sector, multiscale economic teleconnections mitigate or exacerbate water shortages. This study analyzes the spatial and temporal characteristics of Water Footprint (WF) in China from 2005 to 2022 and their relationship with economic development. The findings reveal an overall increase in China’s WF, with the fastest growth observed in the Northwest and Northeast regions. Notably, Heilongjiang, Inner Mongolia, and Xinjiang experienced significant increases, while Zhejiang, Beijing, and Chongqing saw declines. Agricultural water footprint (WF_argi_) account for the largest share, while industrial water footprint (WF_indu_) show a decreasing trend, indicating improved water-use efficiency in industry. However, the rise in contamination water footprint (WF_cont_) and significant spatial disparities in import water footprint (WF_import_) and export water footprint (WF_export_) highlight the challenges in water resources management. Across different regions, there is a strong synergy between agricultural and ecological water use, while a notable trade-offs exists between industrial and agricultural water use, particularly in the Northeast. The relationship between WF consumption and economic development in China exhibits a phased pattern. From 2011 to 2016, most regions achieved strong decoupling, but from 2017 to 2022, all regions reverted to weak decoupling. This study provides new insights into the relationship between sectoral water use and economic development at regional and provincial levels, contributing to efforts to alleviate water scarcity, enhance water resources management, and promote sustainable economic development.

## 1. Introduction

The global water scarcity introduces uncertainties into water resources management, posing challenges to achieving the Sustainable Development Goals (SDGs) [1,2]. Although water scarcity is often perceived as a local issue within river basins, its driving forces and impacts extend across multiple sectors and scales [3]. For instance, agriculture, the sector with the highest water consumption [4,5], frequently trades and consumes products outside of the regions where they are produced [6]. These economic trade connections imply that changes in consumption can significantly impact local water supply systems [7]. As global urbanization leads to an increase of over 2 billion in urban populations by 2030 [8], cities worldwide are expanding rapidly [9]. The negative effects of population concentration and spatial expansion on water ecosystems are becoming increasingly evident, creating dual pressures to enhance high-quality development and the sustainable use of water resources. This challenges the balance between water demand and supply [10] and poses economic risks to water use across sectors, including agriculture, industry, and households [11,12].

Water resources are a critical foundation for economic activities [13,14], and their utilization efficiency and equitable distribution directly impact the achievement of sustainable development. However, significant disparities in water resources use exist across different regions and industries globally, particularly among agricultural, industrial, and ecological water use [15–17]. The competition for water resources is complex and dynamic [12,18]. Martina [19] explored the competition between urban domestic water use and agricultural water use in the context of climate change and urbanization, offering solutions to alleviate water shortages from the perspective of resource competition. Davide [20] examined the competition for water resources driven by large-scale land acquisitions (LSLAs) in the context of economic globalization. The water footprint concept, introduced by Hoekstra (2002), offers a comprehensive measure of direct and indirect water use, encompassing green, blue, and grey water [21]. Spatially, virtual water transfers add complexity to water allocation. For instance, agricultural production often drives interregional water flows, misaligned with local availability [22]. Multi-regional input-output models help quantify trade-offs under water constraints, informing more balanced development strategies [23].

Recent studies increasingly adopt nexus frameworks—especially the Water-Energy-Food (WEF) and Water-Energy-Carbon (WEC) nexuses—to evaluate interactions between water and other key sectors. For example, Petrariu et al. [24] underscored the need for integrated governance in Romania, showing how intersectoral resource flows shape economic and entrepreneurial outcomes. Do et al. [25] used a hydro-economic model in the Lancang-Mekong Basin to demonstrate how adaptive reservoir operations can mitigate trade-offs among hydropower, agriculture, and fisheries. Li et al. [26] highlighted the importance of tracking embodied energy-carbon-water (ECW) flows for low-impact development, while Wang et al. [27] applied ecological network analysis to reveal feedbacks within China’s energy-water system. Together, these findings stress the importance of understanding not just sectoral water quantities, but also their broader economic implications.

Despite significant theoretical and methodological progress in previous studies, there remain two key limitations in current research. First, studies on the synergies and trade-offs in water use have primarily focused on macro-level frameworks—such as resource coupling between water and energy or energy and carbon—while lacking a systematic characterization of sectoral water use relationships within specific domains (e.g., agriculture, industry, and domestic consumption). Second, there is a paucity of empirical analyses linking water resources synergies to economic performance. In light of these gaps, this study focuses on sectoral water use, examining the synergies and trade-offs among different industries, and further exploring their coupling relationships with regional economic development.

This study contributes not only to identifying opportunities for optimizing water use at the micro (sectoral) level but also to developing a macro-level decision-support framework for the coordinated utilization of water resources. By quantitatively examining the interactions between sectoral water use and its economic benefits, the study highlights the balance between improving water resources efficiency and fostering economic growth in China. This approach provides new insights into the complexity of water resources management and offers empirical support for crafting more effective water management policies.

## 2. Data and methods

### 2.1. Study area

China possesses approximately 2.8 trillion cubic meters of total water resources, ranking sixth globally. Nevertheless, its per capita water availability is merely one-fourth of the world average, positioning China among countries experiencing severe water scarcity. The spatial and temporal distribution of water resources in China is highly uneven. Southern regions are endowed with abundant water resources, while northern areas, especially North China and Northwest China, face severe water scarcity and significant interannual variability in precipitation. Major rivers such as the Yangtze River, Yellow River, and Pearl River play crucial roles in supporting economic development and maintaining ecological balance. With rapid industrialization and urbanization, the intensity of water resources exploitation has continued to rise, leading to issues such as water pollution, overexploitation of water resources, and regional water conflicts. Regionally, the eastern part of China has a highly developed economy and high water use intensity, whereas the central and western regions possess relatively abundant resources but have weaker economic bases and lower water use efficiency.

### 2.2. Data acquisition

This study selects the 31 provinces, municipalities, and autonomous regions of China from 2005 to 2022 as the research scope. The results at the regional level were aggregated from those at provincial levels following the geographic regions divisions in China. Based on the definition of WF components in the Water Footprint Assessment Manual [28], this study selects seven sectoral water use indicators: agriculture, industry, domestic, ecology, contamination, import, and export. These indicators account for the total water consumption and pollution processes occurring within a region. The required data includes industrial production, domestic water use, ecological water consumption, agricultural production (including crop yield and livestock production), total import and export value, pollutant emissions, and gross domestic product (GDP). Data sources are the official statistical databases of China, including the following: the Water Resources Bulletin (http://szy.mwr.gov.cn/gbsj/), the China Statistical Yearbook (https://data.cnki.net/Yearbook/), the China Environmental Statistical Yearbook (https://cnki.ctbu.edu.cn/CSYDMirror/trade/Yearbook/Single/), and the National Economic and Social Development Statistical Bulletin (http://www.tjcn.org/tjgb/).

### 2.3. Methods

#### 2.3.1. Accounting for water footprint.

The Water Footprint (WF) is calculated using a bottom-up approach [28], the formula is as follows:


$$WF={WF}_{argi}+{WF}_{indu}+{WF}_{life}+{WF}_{eco}+{WF}_{cont}+{WF}_{import}-{WF}_{export}$$ (1)

In the formula, *WF*_*argi*_ represents the agricultural water footprint, *WF*_*indu*_ represents the industrial water footprint, *WF*_*life*_ represents the domestic water footprint, *WF*_*eco*_ represents the ecological water footprint, *WF*_*cont*_ represents the contamination water footprint, *WF*_*import*_ represents the import water footprint, and *WF*_*export*_ represents the export water footprint.

The contamination water footprint (WF_cont_) [29] refers to the amount of water required to dilute contaminants in wastewater to the concentration levels found in natural water bodies. Due to data limitations and with reference to the research findings of Sun Caizhi et al. [30], the WF_cont_ for chemical oxygen demand (COD) and ammonia nitrogen (NH_3_-N) in industrial wastewater was calculated. The calculation formula is as follows:


$${WF}_{cont-i}=\frac{L_{i}}{C_{i-max}-C_{i-nat}}$$ (2)

$${WF}_{cont}=max\left\{{WF}_{cont-1},{WF}_{cont-2},\ldots ,{WF}_{cont-i},\ldots ,{WF}_{cont-n}\right\}(i=1,2,\ldots ,n)$$ (3)

In the formula, *WF*_*cont-i*_ represents the water footprint of contaminant *i*; *L* is the contaminant load, measured in kg/a; *C*_*max*_ is the maximum acceptable mass concentration of the contaminant, measured in kg/m^3^; *C*_*nat*_ is the natural mass concentration of the contaminant in the water body, measured in kg/m³. The value for *C*_*max*_ is based on the Class IV pollutant standard mass concentration specified by the Environmental Quality Standards for Surface Water in China (GB 3838–2002), and *C*_*nat*_ is set to 0.

#### 2.3.2. Calculation of synergies and trade-offs in sectoral water use.

Spearman correlation analysis is a non-parametric statistical method. Unlike the Pearson correlation coefficient, the Spearman correlation coefficient does not require data to be linearly related or normally distributed, making it suitable for handling non-linear relationships or data with outliers. The longitudinal Spearman correlation analysis covering non-linear relations were conducted on all water use indicators across China’s 6 geographical divisions and 31 provinces. To account for multiple correlation tests, a Bonferroni correction was applied to adjust the p-values [31]. The Bonferroni correction adjusts the significance threshold by dividing the original alpha level (e.g., 0.05) by the number of comparisons, thereby providing a conservative yet statistically robust control for false positives in large-scale correlation matrices. This ensures that the relationships between water use indicators are statistically meaningful and not due to random variation. Furthermore, indicator pairs were selected where the absolute value of the correlation coefficient |R| exceeded 0.6. The Spearman correlation coefficient is calculated using the following formula:


$$R=1-\frac{6d_{i}^{2}}{n\left(n^{2}-1\right)}$$ (4)

In the formula, *n* is the number of data points, and *d*_*i*_ is the rank difference for the *i*^*th*^ data point. According to widely recognized guidelines [32], a correlation coefficient between 0.70 and 0.90 is generally considered “strong.” We selected a relatively high threshold of 0.82 to ensure that only relationships with a very robust association are labeled as strong synergies or trade-offs. This threshold helps to minimize the influence of spurious correlations, particularly given the large number of statistical tests performed, and it is consistent with prior studies in the field of water resources management [23,33]. The synergies and trade-offs are categorized into four levels: Synergy (Bonferroni p < 0.05, ABS(R)>0.82), Weak Synergy (Bonferroni p>=0.05, ABS(R)<=0.82), Weak Trade-off (Bonferroni p>=0.05, ABS(R)<=0.82), Trade-off (Bonferroni p < 0.05, ABS(R)>0.82). ABS(R): Absolute value of R.

Partial correlation analysis measures the linear relationship between two variables while controlling for the linear effects of other variables. When the effect of one variable is controlled, the resulting value is called the first-order partial correlation coefficient. When the effects of two variables are controlled, the resulting value is called the second-order partial correlation coefficient. The formula is as follows:


$$\begin{array}{c} r_{xy\cdot z}=\frac{r_{xy}-r_{xz}r_{yz}}{\sqrt{\left(1-r_{xz}^{2}\right)\left(1-r_{yz}^{2}\right)}} \end{array}$$ (5)

where *r*_*xy∙z*_ epresents the first-order partial correlation coefficient between variables *x* and *y* after controlling for the effect of variable *z.*

The second-order partial correlation coefficient is derived from *r*_*xy∙z*_, with the formula:


$$\begin{array}{c} r_{ij\cdot hm}=\frac{r_{ij\cdot h}-r_{im\cdot h}r_{jm\cdot h}}{\sqrt{\left(1-r_{im\cdot h}^{2}\right)\left(1-r_{jm\cdot h}^{2}\right)}} \end{array}$$ (6)

Where *r*_*ij∙jm*_ represents the partial correlation coefficient between variables *i* and *j* after controlling for the effect of variable *h* and *m.*

Finally, the significance of the second-order partial correlation coefficient needs to be tested. In this study, the t-test method is employed, with the formula as follows:


$$\begin{array}{c} t=\frac{r_{ij\cdot hm}}{\sqrt{1-r_{ij\cdot hm}^{2}}}\sqrt{n-m-1} \end{array}$$ (7)

where *r*_*ij∙jm*_ is the second-order partial correlation coefficient, *n* is the sample size, and *m* is the number of degrees of freedom.

#### 2.3.3. Calculation of coordinated relationships based on the theory of decoupling.

The Tapio decoupling theory model [34] uses the elasticity decoupling index to measure the degree of decoupling, commonly employed to study the relationship between resource and environmental use and economic growth. Specifically, when the elasticity index is between 0 and 0.8, it indicates weak decoupling; between 0.8 and 1.2, it is classified as expansive decoupling; and greater than 1.2 indicates expansive negative decoupling [35–37]. This paper establishes a decoupling model based on WF and regional GDP to assess the decoupling relationship between WF and regional GDP. The calculation formula is:


$$D_{e}=\frac{\Delta W_{F}}{\Delta GDP}=\frac{\frac{\left(W_{F,t+1}-W_{F,t}\right)}{W_{F,t}}}{\frac{{GDP}_{t+1}-{GDP}_{t}}{{GDP}_{t}}}$$ (8)

where: *D*_*e*_ is Decoupling index; *W*_*F*_ is Water footprint (total water footprint across sectors), m^3^; *GDP* is Gross Domestic Product, yuan; *ΔW*_*F*_ is Change rate in water footprint; *ΔGDP* is Change rate in regional GDP; *t* is Starting year of calculation; *t + 1* is Ending year of calculation.

## 3. Results

### 3.1. Temporal and spatial distribution characteristics of total water footprint

**Fig 1:**
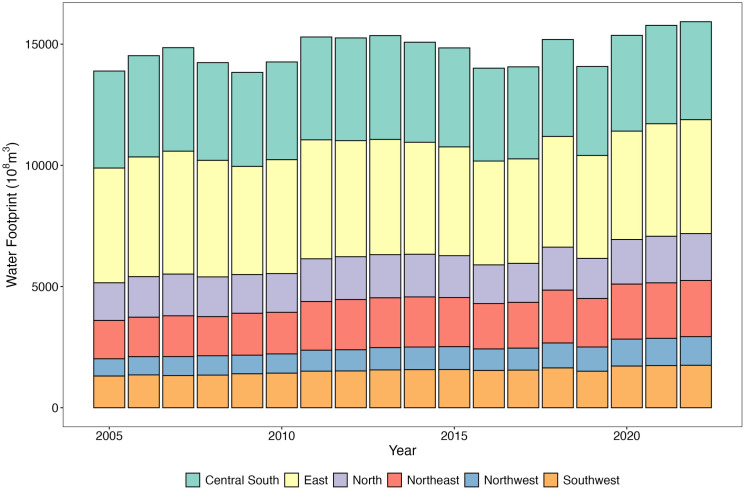
Water footprint at regional levels in China.

**Fig 2:**
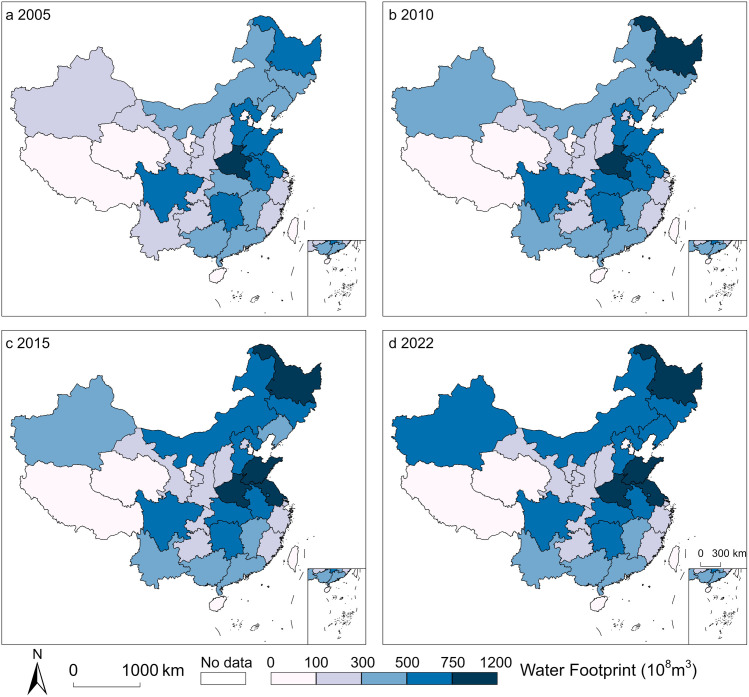
The distribution of water footprint levels in China. a, 2005. b, 2010. c, 2015. d, 2022. The basemap of China was obtained from the Standard Map Service of the Ministry of Natural Resources of the People’s Republic of China (Review Number: GS(2019)1822). The map is used for academic purposes. All rights reserved by the Ministry of Natural Resources. (Available at: http://bzdt.ch.mnr.gov.cn/).

Comparing the total water footprint (WF) across different regions of China from 2005 to 2022 (Fig 1), and using the natural breaks method to classify the WF into five levels (Fig 2). At the regional level, the Northwest and Southwest regions have the largest WF. From west to east, the WF and its average value show an upward trend, with an increasing gap between the maximum and minimum values. Overall, from 2005 to 2022, the WF in various regions has increased, with the Northwest and Northeast regions experiencing the fastest growth rates of 72.46% and 59.71%, respectively. This is due to the Northwest and Northeast regions being major suppliers of agricultural and livestock products in China, where agriculture is the largest water-using sector. Especially in areas with limited water resources and precipitation, the demand for irrigation places significant pressure on water resources. At the provincial level, Heilongjiang and Henan consistently have high WF, with average annual WF of 892.66 × 10^8^m^3^ and 887.07 × 10^8^m^3^, respectively. In contrast, Tibet and Qinghai have low WF, with average annual WF of 35.71 × 10^8^m^3^ and 42.10 × 10^8^m^3^, respectively. Heilongjiang, Inner Mongolia, and Xinjiang have experienced significant increases in WF, with growth rates of 530.97%, 339.60%, and 256.49%, respectively, from 2005 to 2022. Conversely, Zhejiang, Beijing, and Chongqing have seen reductions in WF consumption, with decreases of 54.83%, 23.75%, and 17.34%, respectively.

### 3.2. Temporal and spatial distribution characteristics of sectoral water footprint

**Fig 3:**
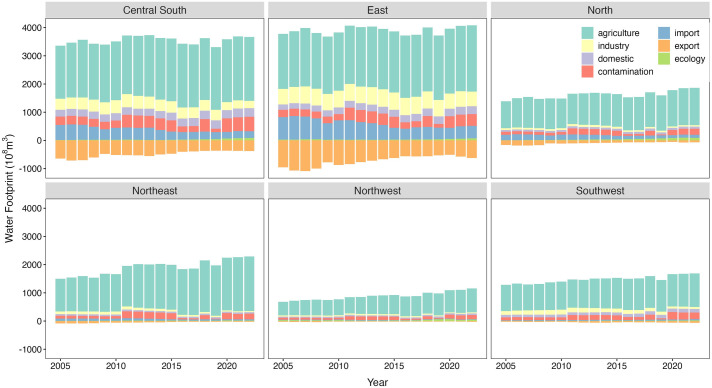
Characteristics of water footprint by sector at the regional level in China. Note: To better illustrate the differences in water footprint across various sectors in each province, we have replaced outliers with the upper quartile values for each sector in this figure.

As an important agricultural production region, China’s agricultural water footprint (WF_argi_) is the largest (Fig 3). The proportion and increase of contamination water footprint (WF_cont_) exceed those of ecological water footprint (WF_eco_), indicating that while the demand for ecological water in rivers, lakes, and wetlands is increasing, urbanization and the scale of agricultural production are also expanding. At the regional level, except for the Eastern and Central South regions where the proportion of WF_argi_ remains stable, the remaining four regions show an increasing trend. This suggests that as agricultural water demand grows, there is a need to enhance water-saving measures and improve water use efficiency in irrigation areas. The import water footprint (WF_import_) and export water footprint (WF_export_) show significant spatial differences, with the southeastern coastal regions having noticeably higher WF compared to the northwestern inland regions.

**Fig 4:**
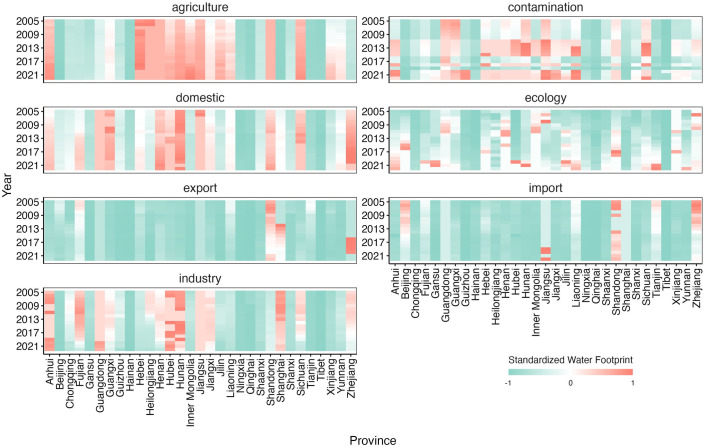
Characteristics of water footprint by sector at the provincial level.

At the provincial level (Fig 4), Sichuan, Anhui, and Inner Mongolia have high proportions of WF_argi_ with an increasing trend. Hebei also has a high proportion but shows a decreasing trend, while Shanghai, Hainan, Beijing, Qinghai, and Tibet have low proportions. Hunan, Zhejiang, Henan, and Guangxi have higher domestic water footprint (WF_life_), while Ningxia, Qinghai, and Tibet have lower WF_life_. Hubei, Hunan, Anhui, and Shanghai exhibit higher WF_indu_, whereas Hainan, Ningxia, Tibet, and Qinghai have lower WF_indu_. WF_import_ is generally low, with slightly higher values in Shandong, Zhejiang, and Jiangsu. WF_export_ is also generally low, with Shandong, Shanghai, and Zhejiang showing slightly higher values. The WF_cont_ shows significant temporal variation, with a rapid increase from 2010 to 2015, followed by a slowdown after 2015. Hunan, Jiangsu, Liaoning, and Sichuan have higher WF_cont_, while Tibet and Qinghai have lower values. Henan and Shandong have higher WF_eco_, while Tibet and Hainan have lower WF_eco_.

### 3.3. Synergies and trade-offs in sectoral water use

**Fig 5:**
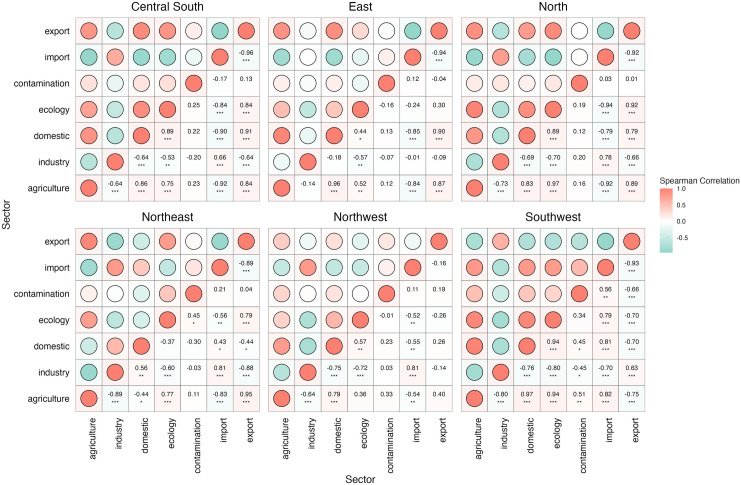
Synergy and trade-offs of sectoral water use at the regional level in China. Note: ***, **, and * denote p-values < 0.01, < 0.05, and < 0.1, respectively.

At the regional level (Fig 5), among 126 pairs of indicators, 17 had synergistic effects and 15 had trade-offs effects, with ABS (R) of 0.87–0.95 and 0.88–0.93. East and Southwest showed the strongest synergistic effect in agriculture and domestic water use, with ABS (R) of 0.96 and 0.97. The strongest trade-offs in different regions are different: export and ecology water use in Central South (ABS (R)=0.96), import and domestic water use in East (ABS (R)=0.94), agriculture and ecology water use in the North (ABS (R)=0.94), industry and agriculture water use in the Northeast (ABS (R)=0.89), industry and domestic water use in Northwest (ABS (R)=0.75) and ecology and domestic water use in Southwest (ABS (R)=0.93). In general, synergies/weak synergies are generally shown in ecology and agriculture in China, with an average ABS (R) of 0.72, and synergies in North (ABS (R)=0.97) and Southwest (ABS (R)=0.94) significantly higher than those in other regions. In the industry and agriculture water use, the average ABS (R) was 0.64; in the Northeast (ABS (R)=0.89), the trade-offs was significantly higher than that in other regions. At the same time, weak trade-offs were shown in industry and ecology water use, with an average ABS (R) of 0.65.

**Fig 6:**
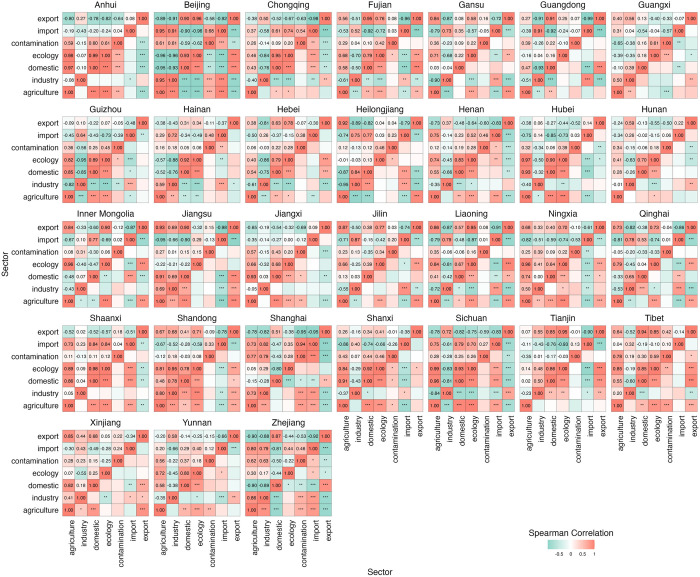
Synergy and trade-offs of sectoral water use at the provincial level. Note: ***, **, and * denote p-values < 0.01, < 0.05, and < 0.1, respectively.

At the provincial level (Fig 6), there are differences in sectoral synergies and trade-offs within China. Most sectoral water use interactions exhibit higher levels of synergy than trade-offs. Among 650 pairs of indicators, 60 pairs show synergy effects and 52 pairs show trade-offs, with average ABS(R) of 0.83–0.98 and 0.82–0.94 (Bonferroni corrected for p < 0.05 and ABS(R)>0.6). 10 provinces showed the strongest synergies between domestic and ecology water use, 5 provinces showed the strongest synergies between agriculture and ecology water use, ABS (R) was 0.70–0.99 and 0.79–0.99. 13 provinces showed the strongest trade-offs between import and export water use, and 5 provinces showed the strongest trade-offs between industry and ecology water use, with ABS (R) of 0.74–0.99 and 0.63–0.94. The synergistic effect in Anhui is the highest in domestic and ecology water use, while that in Sichuan is the highest in agriculture and ecology water use. Guangdong has the strongest trade-offs effect between import and export water use, while Beijing has the strongest trade-offs effect between industry and ecology water use.

**Fig 7:**
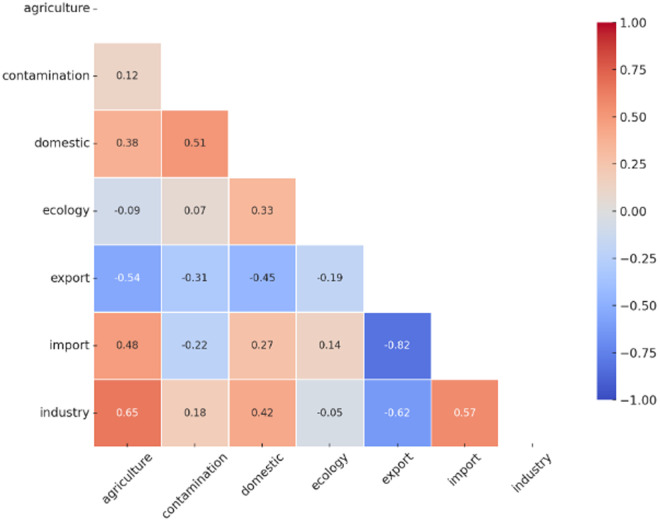
Second-order partial correlation analysis of sectoral water use in China from 2005 to 2022.

The second-order partial correlation analysis reveals significant relationships in water use between different sectors in China after controlling for overall water use trends (Fig 7). A strong positive correlation was found between agricultural and industrial water use (r = 0.65, p = 0.001), indicating close sectoral linkage, possibly due to agricultural processing and shared infrastructure. Agriculture also showed a positive relationship with import water use (r = 0.48, p = 0.023), suggesting reliance on imported virtual water.

Conversely, agriculture and export water use were negatively correlated (r = –0.54, p = 0.008), highlighting a potential trade-offs, possibly due to large volumes of virtual water embodied in exported goods. Domestic water use was moderately correlated with agriculture (r = 0.38, p = 0.067) and significantly with both contamination (r = 0.51, p = 0.015) and industry (r = 0.42, p = 0.042), reflecting urban water stress. A strong negative correlation was found between export and import water use (r = –0.82, p < 0.001), while import and industry were positively related (r = 0.57, p = 0.005), pointing to the industrial sector’s increasing dependence on imported virtual water. No significant correlations were observed between ecological water use and other types, suggesting limited integration of ecological needs in broader water use patterns.

### 3.4. Coordination Based on the Decoupling Theory Model

**Fig 8:**
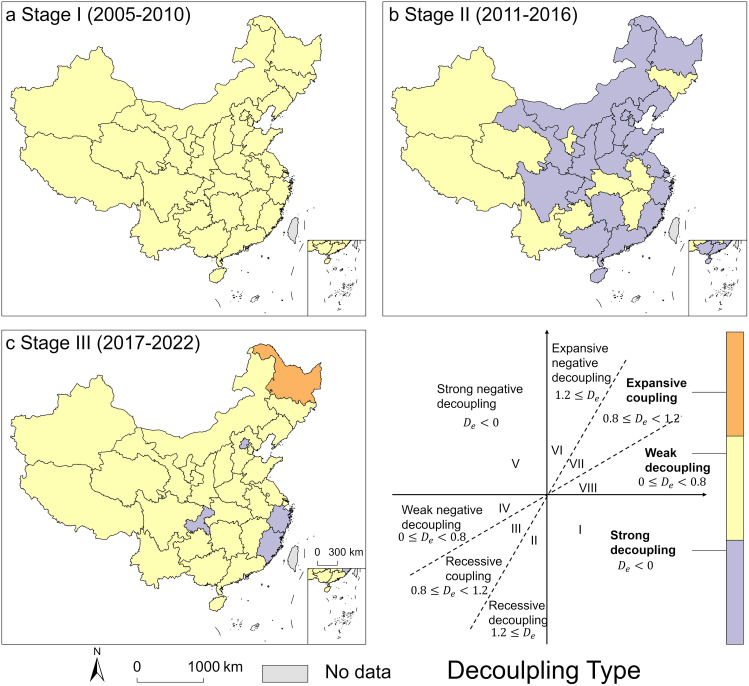
Decoupling index of water footprint and GDP at the provincial level. *The basemap of China was obtained from the Standard Map Service of the Ministry of Natural Resources of the People’s Republic of China (Review Number: GS(2019)1822). The map is used for academic purposes. All rights reserved by the Ministry of Natural Resources.* (Available at: *http://bzdt.ch.mnr.gov.cn/).*

Due to significant changes in China’s WF around 2011 and 2016, the study period was divided into three stages, with 2011 and 2016 serving as inflection points: Stage I (2005–2010), Stage II (2011–2016), and Stage III (2017–2022). The decoupling index for each stage is presented in Table 1 and Fig 8.

**Table 1:** Decoupling index of water footprint and GDP at the regional level in China.

Period	Central South	East	North	Northeast	Northwest	Southwest
Stage I (2005-2010)	Weak decoupling	Weak decoupling	Weak decoupling	Weak decoupling	Weak decoupling	Weak decoupling
Stage II (2011-2016)	Strong decoupling	Strong decoupling	Strong decoupling	Strong decoupling	Weak decoupling	Weak decoupling
Stage III (2017-2022)	Weak decoupling	Weak decoupling	Weak decoupling	Weak decoupling	Weak decoupling	Weak decoupling

As seen in Table 1, during Stage I, all regions of China experienced weak decoupling, where the growth rate of the WF was lower than the growth rate of regional GDP, indicating a state of relatively coordinated development. In Stage II, the Central South, East, North, and Northwest regions exhibited strong decoupling, with a reduction in the WF accompanied by an increase in regional GDP, indicating a state of coordinated development. This suggests that these regions had effective control over water usage relative to economic growth. The remaining regions continued to show weak decoupling, still in a relatively coordinated development state. In Stage III, all regions exhibited weak decoupling, with the WF and regional GDP remaining in a relatively coordinated state, though still falling short of the strong decoupling target.

At the provincial level, as shown in Fig 8a, all provinces exhibited weak decoupling during Stage I, indicating a relatively coordinated development state between WF and regional GDP. In Stage II, as depicted in Fig 8b, most regions demonstrated strong decoupling, with a reduction in the WF alongside economic growth, indicating coordinated development. However, Jilin, Anhui, Jiangxi, Hubei, Guizhou, Yunnan, Ningxia, Qinghai, Xinjiang, and Tibet continued to show weak decoupling, remaining in a relatively coordinated development state between WF and regional GDP. In Stage III, as shown in Fig 8c, Beijing, Zhejiang, Fujian, and Chongqing exhibited strong decoupling, indicating coordinated development between WF and regional GDP. Heilongjiang showed an expansive coupling, where both the WF and regional GDP grew in tandem, indicating low water resources use efficiency and significant environmental pressure due to economic growth. The remaining regions exhibited weak decoupling, where the WF grew at a slower pace than regional GDP, indicating a relatively coordinated development state.

## 4. Discussion

From the perspective of the WF, we utilized Spearman correlation coefficient and the decoupling theory model to analyze the synergies and trade-offs in sectoral water use, as well as its coordination with economic development. By constructing a sectoral water use interaction network and using a unified dataset of 776 indicators at the regional and provincial levels in China, we quantitatively and systematically identified the synergy and economic benefits of water use across seven different sectors. This understanding enhances insights into the complexity and importance of water resources management, improving water use efficiency and balancing the conflict between economic development and ecological protection [38]. As a result, it can reduce water resources conflicts, promote sustainable economic growth, and contribute to achieving broader sustainable development goals.

### 4.1. Water footprint changes and driving mechanisms

The increase in WF reflects China’s rapid economic growth and rising consumption demands, particularly in meat consumption and industrial expansion, which have led to higher water resources consumption. Climate change-induced extreme weather events affect the availability and distribution of water resources, increasing the difficulty of water resources management and resulting in changes in the WF [39]. Although agriculture occupies a large share of the WF, the decline in WF_indu_ reflects advancements in water resources management, technological innovation, industrial restructuring, and environmental protection efforts within the industrial sector [40]. This trend not only alleviates water resources pressure but also promotes sustainable economic development. However, the increase in WF_cont_ indicates that contamination from industrial and agricultural sectors remains a challenge, especially with improper wastewater treatment and chemical discharges [41].

Increased agricultural water use often accompanies a rise in ecological water use, particularly in northern arid regions and the ecologically fragile Southwest [42,43]. In water-scarce northern areas, rising agricultural water use can significantly squeeze ecological water use, exacerbating ecological stress. In the Southwest, the complex terrain makes ecosystems more sensitive to agricultural water extraction.

### 4.2. Dynamics of synergies and trade-offs in sectoral water use

The trade-offs between industrial and agricultural water use in China indicates a negative correlation between these sectors in different regions. This negative correlation reflects the complementary or substitutive nature of industrial and agricultural sectors across different regions of China. It also reveals that there may be competition for water resources between agriculture and industry in certain areas. When water resources are prioritized for industrial development, agricultural water use may be restricted, leading to a decrease in agricultural water consumption [44]. The competition between industrial and agricultural water use is most pronounced in the Northeast China. As a traditional industrial and agricultural hub, competition over water resources intensifies during agricultural peak seasons, raising challenges for wetland conservation and water management [45].

Meanwhile, the weak trade-offs between industrial and ecological water use indicates that although competition exists, its intensity remains limited. Effective integrated water resources management and technological innovation have mitigated potential ecological pressures [46,47]. Balancing the needs of agricultural development and ecological protection is a critical issue in China’s water resources management. Since agricultural activities generally have high water demands, especially for irrigation, an increase in agricultural water use can lead to higher ecological water needs.

### 4.3. Decoupling trends between water footprint and economic growth

Driven by rapid economic growth and industrialization, China exhibited weak decoupling across all regions during Stage I (2005–2010). With an average annual GDP growth rate of around 10%, the country experienced accelerated industrialization, urbanization, and large-scale infrastructure development. These activities significantly increased water resources demand. In Stage II (2011–2016), most regions achieved strong decoupling. This improvement was supported by the release of the *National Water Resources Comprehensive Planning* in 2011 and the 12th Five-Year Plan’s emphasis on building a water-saving society. However, some central, western, and northeastern regions continued to show weak decoupling due to their weaker economic foundations, reliance on resource-intensive industries, and underdeveloped water management infrastructure, leading to a rapid increase in WF.

During Stage III (2017–2022), all regions returned to a weak decoupling state amid China’s transition to a “new economic normal” and increasing environmental pressures. After 2017, GDP growth slowed to around 6%, and environmental protection measures were strengthened under the 13th Five-Year Plan, which promoted green development. Nevertheless, continued dependence on traditional industries and challenges in advancing water-saving technologies limited progress. For example, while northern regions improved water pollution control and optimized water use structures [48], the high concentration of population and industry made rapid WF reduction difficult. In the southwest, complex geography and challenging water management also constrained declines in WF.

## 5. Conclusion

(1) From 2005 to 2022, China’s water footprint exhibited an overall increasing trend, with the Northwest and Northeast regions experiencing the fastest growth. Agricultural water use remained dominant, industrial WF declined, but contamination-related WF continued to rise. Climate change, economic restructuring, and policy interventions jointly drove the spatiotemporal evolution of the WF.
(2) A significant synergy was found between agricultural water use and ecological water needs. There was a notable trade-offs between industrial and agricultural water use, while the competition between industrial and ecological water use was relatively weak. Strong synergies were observed between agricultural and ecological water use in northern and southwestern regions, while significant trade-offs between industrial and agricultural water use were most evident in the Northeast. However, some trade-offs, particularly in virtual water imports and exports, still require careful management.
(3) China experienced a transition from weak decoupling to strong decoupling, and then back to weak decoupling over the study period. The eastern region showed significant decoupling, while water footprint in the central and western regions remained closely tied to economic growth, highlighting substantial regional differences in decoupling progress.

## Supporting information

S1 File Supporting information. (XLSX)

## References

[1] van Vliet MTH, van Beek LPH, Eisner S, Flörke M, Wada Y, Bierkens MFP. Multi-model assessment of global hydropower and cooling water discharge potential under climate change. Global Environ Change. 2016;40:156–70. doi: 10.1016/j.gloenvcha.2016.07.007

[2] Global risk report 2020. https://www.weforum.org/publications/the-global-risks-report-2020/

[3] Vörösmarty CJ, Hoekstra AY, Bunn SE, Conway D, Gupta J. WATER. Fresh water goes global. Science. 2015;349(6247):478–9. doi: 10.1126/science.aac6009 26228130

[4] Hoekstra AY, Mekonnen MM. The water footprint of humanity. Proc Natl Acad Sci U S A. 2012;109(9):3232–7. doi: 10.1073/pnas.1109936109 22331890 PMC3295316

[5] Gleick PH. The World’s Water. Washington, DC: Island Press/Center for Resource Economics; 2014. doi: 10.5822/978-1-61091-483-3

[6] Dalin C, Konar M, Hanasaki N, Rinaldo A, Rodriguez-Iturbe I. Evolution of the global virtual water trade network. Proc Natl Acad Sci U S A. 2012;109(16):5989–94. doi: 10.1073/pnas.1203176109 22474363 PMC3341016

[7] Marston L, Konar M, Cai X, Troy TJ. Virtual groundwater transfers from overexploited aquifers in the United States. Proc Natl Acad Sci U S A. 2015;112(28):8561–6. doi: 10.1073/pnas.1500457112 26124137 PMC4507249

[8] 2014 revision of the World Urbanization Prospects. United Nations; 2014. https://www.un.org/en/development/desa/publications/2014-revision-world-urbanization-prospects.html

[9] Jiang L, O’Neill BC. Global urbanization projections for the Shared Socioeconomic Pathways. Global Environ Change. 2017;42:193–9. doi: 10.1016/j.gloenvcha.2015.03.008

[10] Li J, Liu Z, He C, Yue H, Gou S. Water shortages raised a legitimate concern over the sustainable development of the drylands of northern China: Evidence from the water stress index. Sci Total Environ. 2017;590–591:739–50. doi: 10.1016/j.scitotenv.2017.03.037 28284646

[11] Flörke M, Kynast E, Bärlund I, Eisner S, Wimmer F, Alcamo J. Domestic and industrial water uses of the past 60 years as a mirror of socio-economic development: a global simulation study. Global Environ Change. 2013;23(1):144–56. doi: 10.1016/j.gloenvcha.2012.10.018

[12] Dolan F, Lamontagne J, Link R, Hejazi M, Reed P, Edmonds J. Evaluating the economic impact of water scarcity in a changing world. Nat Commun. 2021;12(1):1915. doi: 10.1038/s41467-021-22194-0 33772023 PMC7997906

[13] Moss RH, Edmonds JA, Hibbard KA, Manning MR, Rose SK, van Vuuren DP, et al. The next generation of scenarios for climate change research and assessment. Nature. 2010;463(7282):747–56. doi: 10.1038/nature08823 20148028

[14] Hanasaki N, Fujimori S, Yamamoto T, Yoshikawa S, Masaki Y, Hijioka Y, et al. A global water scarcity assessment under Shared Socio-economic Pathways – Part 1: Water use. Hydrol Earth Syst Sci. 2013;17(7):2375–91. doi: 10.5194/hess-17-2375-2013

[15] Zhao X, Liu J, Yang H, Duarte R, Tillotson MR, Hubacek K. Burden shifting of water quantity and quality stress from megacity Shanghai. Water Resources Res. 2016;52(9):6916–27. doi: 10.1002/2016wr018595

[16] Gleeson T, Wada Y, Bierkens MFP, van Beek LPH. Water balance of global aquifers revealed by groundwater footprint. Nature. 2012;488(7410):197–200. doi: 10.1038/nature11295 22874965

[17] Gleick PH. Global freshwater resources: soft-path solutions for the 21st century. Science. 2003;302(5650):1524–8. doi: 10.1126/science.1089967 14645837

[18] OECD. OECD environmental outlook to 2050: the consequences of inaction. Paris: Organisation for Economic Co-operation and Development; 2012. https://www.oecd-ilibrary.org/environment/oecd-environmental-outlook-to-2050_9789264122246-en

[19] Flörke M, Schneider C, McDonald RI. Water competition between cities and agriculture driven by climate change and urban growth. Nat Sustain. 2018;1(1):51–8. doi: 10.1038/s41893-017-0006-8

[20] Chiarelli DD, D’Odorico P, Müller MF, Mueller ND, Davis KF, Dell’Angelo J, et al. Competition for water induced by transnational land acquisitions for agriculture. Nat Commun. 2022;13(1):505. doi: 10.1038/s41467-022-28077-2 35082300 PMC8791946

[21] Hoekstra AY, Chapagain AK. Water footprints of nations: Water use by people as a function of their consumption pattern. Water Resour Manage. 2006;21(1):35–48. doi: 10.1007/s11269-006-9039-x

[22] Zhang Z, Yang H, Shi M. Spatial and sectoral characteristics of China’s international and interregional virtual water flows – based on multi-regional input–output model. Econ Syst Res. 2016;28(3):362–82. doi: 10.1080/09535314.2016.1165651

[23] Zhao D, Liu J, Sun L, Ye B, Hubacek K, Feng K, et al. Quantifying economic-social-environmental trade-offs and synergies of water-supply constraints: an application to the capital region of China. Water Res. 2021;195:116986. doi: 10.1016/j.watres.2021.116986 33721677

[24] Petrariu R, Constantin M, Dinu M, Pătărlăgeanu SR, Deaconu ME. Water, energy, food, waste nexus: between synergy and trade-offs in romania based on entrepreneurship and economic performance. Energies. 2021;14(16):5172. doi: 10.3390/en14165172

[25] Do P, Tian F, Zhu T, Zohidov B, Ni G, Lu H, et al. Exploring synergies in the water-food-energy nexus by using an integrated hydro-economic optimization model for the Lancang-Mekong River basin. Sci Total Environ. 2020;728:137996. doi: 10.1016/j.scitotenv.2020.137996 32570321

[26] Li H, Zhao Y, Kang J, Wang S, Liu Y, Wang H. Identifying sectoral energy-carbon-water nexus characteristics of China. J Cleaner Production. 2020;249:119436. doi: 10.1016/j.jclepro.2019.119436

[27] Wang S, Fath B, Chen B. Energy–water nexus under energy mix scenarios using input–output and ecological network analyses. Appl Energy. 2019;233–234:827–39. doi: 10.1016/j.apenergy.2018.10.056

[28] Egan M. The water footprint assessment manual. setting the global standard. Soc Environ Accountability J. 2011;31(2):181–2. doi: 10.1080/0969160x.2011.593864

[29] Cai J, He Y, Xie R, Liu Y. A footprint-based water security assessment: An analysis of Hunan province in China. J Cleaner Production. 2020;245:118485. doi: 10.1016/j.jclepro.2019.118485

[30] Sun C, Liu Y, Chen L, Zhang L. The spatial-temporal disparities of water footprints intensity based on Gini coefficient and Theil index in China. Acta Ecologica Sinica. 2010;30:1312–21.

[31] Wolfram MathWorld: The Web’s Most Extensive Mathematics Resource. Accessed 2024 August 14 https://mathworld.wolfram.com/

[32] Hinkle DE, Wiersma W, Jurs SG. Applied statistics for the behavioral sciences. Boston: Houghton Mifflin; 1988.

[33] Wu L, Elshorbagy A, Pande S, Zhuo L. Trade-offs and synergies in the water-energy-food nexus: the case of Saskatchewan, Canada. Resources Conservation Recycling. 2021;164:105192. doi: 10.1016/j.resconrec.2020.105192

[34] Tapio P. Towards a theory of decoupling: degrees of decoupling in the EU and the case of road traffic in Finland between 1970 and 2001. Transport Policy. 2005;12(2):137–51. doi: 10.1016/j.tranpol.2005.01.001

[35] Sun J, Sun S, Yin Y, Wang Y, Zhao J, Tang Y, et al. Decoupling trend and drivers between grain water‑carbon footprint and economy-ecology development in China. Agric Syst. 2024;217:103904. doi: 10.1016/j.agsy.2024.103904

[36] Zhang Y, Sun M, Yang R, Li X, Zhang L, Li M. Decoupling water environment pressures from economic growth in the Yangtze River Economic Belt, China. Ecol Indicators. 2021;122:107314. doi: 10.1016/j.ecolind.2020.107314

[37] Lu N, Zhu J, Tang Z, Zhang J, Chi H. Decreasing water dependency for economic growth in water-scarce regions by focusing on water footprint and physical water: a case study of Xi’an, China. Sustainable Cities Soc. 2022;85:104092. doi: 10.1016/j.scs.2022.104092

[38] Strokal M, Kahil T, Wada Y, Albiac J, Bai Z, Ermolieva T, et al. Cost-effective management of coastal eutrophication: a case study for the Yangtze river basin. Resources Conservation Recycling. 2020;154:104635. doi: 10.1016/j.resconrec.2019.104635

[39] Chukalla AD, Krol MS, Hoekstra AY. Marginal cost curves for water footprint reduction in irrigated agriculture: guiding a cost-effective reduction of crop water consumption to a permit or benchmark level. Hydrol Earth Syst Sci. 2017;21(7):3507–24. doi: 10.5194/hess-21-3507-2017

[40] Fan J-L, Wang J-D, Zhang X, Kong L-S, Song Q-Y. Exploring the changes and driving forces of water footprints in China from 2002 to 2012: a perspective of final demand. Sci Total Environ. 2019;650(Pt 1):1101–11. doi: 10.1016/j.scitotenv.2018.08.426 30308798

[41] Ma T, Sun S, Fu G, Hall JW, Ni Y, He L, et al. Pollution exacerbates China’s water scarcity and its regional inequality. Nat Commun. 2020;11(1):650. doi: 10.1038/s41467-020-14532-5 32005847 PMC6994511

[42] Wang H, Qin L, Huang L, Zhang L. Ecological agriculture in China: principles and applications. In: Sparks DL, ed. Advances in Agronomy. Academic Press; 2007: 181–208. doi: 10.1016/s0065-2113(06)94004-8

[43] Hou J, Zhang M, Li Y. Can digital economy truly improve agricultural ecological transformation? New insights from China. Humanit Soc Sci Commun. 2024;11(1). doi: 10.1057/s41599-023-02593-y

[44] Shah WUH, Hao G, Yasmeen R, Yan H, Qi Y. Impact of agricultural technological innovation on total-factor agricultural water usage efficiency: Evidence from 31 Chinese Provinces. Agric Water Manag. 2024;299:108905. doi: 10.1016/j.agwat.2024.108905

[45] Fang Z, Luo N, Chiu Y-H. Sustainable efficiency in cities in China-An interaction model among water, energy, and industry. Sci Total Environ. 2024;927:172154. doi: 10.1016/j.scitotenv.2024.172154 38575029

[46] Zhang C-Y, Oki T. Water pricing reform for sustainable water resources management in China’s agricultural sector. Agric Water Manag. 2023;275:108045. doi: 10.1016/j.agwat.2022.108045

[47] Yang P, Zhu Y, Zhai X, Xia J, Chen Y, Huang H, et al. Adaptive management of water resources system in the arid Aksu river basin, northwest China. J Cleaner Production. 2023;419:138185. doi: 10.1016/j.jclepro.2023.138185

[48] Luo Z, Ji L, Xie Y. Multi-dimensional equilibrium optimal allocation of water resources in Beijing-Tianjin-Hebei region through spatial association network analysis. J Hydrol. 2024;642:131826. doi: 10.1016/j.jhydrol.2024.131826

